# Excitation Conditions for Surface-Enhanced Hyper Raman Scattering With Biocompatible Gold Nanosubstrates

**DOI:** 10.3389/fchem.2021.680905

**Published:** 2021-05-17

**Authors:** Arpad Dusa, Fani Madzharova, Janina Kneipp

**Affiliations:** Department of Chemistry, Humboldt-Universität zu Berlin, Berlin, Germany

**Keywords:** gold nanoparticles, gold nanorods, finite-difference time-domain, electromagnetic enhancement, dimer, surface-enhanced hyper Raman scattering, surface-enhanced Raman scattering, two-photon excitation

## Abstract

Surface enhanced hyper Raman scattering (SEHRS) can provide many advantages to probing of biological samples due to unique surface sensitivity and vibrational information complementary to surface-enhanced Raman scattering (SERS). To explore the conditions for an optimum electromagnetic enhancement of SEHRS by dimers of biocompatible gold nanospheres and gold nanorods, finite-difference time-domain (FDTD) simulations were carried out for a broad range of excitation wavelengths from the visible through the short-wave infrared (SWIR). The results confirm an important contribution by the enhancement of the intensity of the laser field, due to the two-photon, non-linear excitation of the effect. For excitation laser wavelengths above 1,000 nm, the hyper Raman scattering (HRS) field determines the enhancement in SEHRS significantly, despite its linear contribution, due to resonances of the HRS light with plasmon modes of the gold nanodimers. The high robustness of the SEHRS enhancement across the SWIR wavelength range can compensate for variations in the optical properties of gold nanostructures in real biological environments.

## Introduction

Hyper Raman scattering (HRS), a two-photon excited spontaneous Raman process, analogous to its one-photon counterpart Raman scattering, can be enhanced in the vicinity of a metal nanostructure, as surface-enhanced hyper Raman scattering (SEHRS) (Baranov and Bobovich, 1982; Madzharova et al., 2017). In SEHRS, the very inefficient process of HRS can reach two-photon cross sections corresponding to 10^4^–10^5^ GM, making it easily competitive with two-photon fluorescence probing, also in biological samples (Kneipp et al., 2006a). The many benefits of two-photon excitation include deep penetration and high biocompatibility due to long wavelengths of the excitation light, detection in the visible wavelength range, and strong localization of the probing. In SEHRS they are combined with the high enhancement, the nanometer-scaled confinement, and with the unique ability of HRS to probe molecular vibrations that do not appear in Raman spectra, or even of silent modes observed neither by Raman nor IR spectroscopy (Kneipp et al., 2006b), due to different selection rules of the process that relies on a change in the hyperpolarizability of a molecule (Ziegler, 1990). This results in extraordinary possibilities to study e.g., the interaction of an organic or biological molecule with a surface (Golab et al., 1988; Hulteen et al., 2006).

Enhancement in SEHRS involves both, a chemical enhancement (Golab et al., 1988; Valley et al., 2010), that can become very important depending on the system that is probed, and an efficient electromagnetic enhancement (Kneipp et al., 1999), due to the non-linearity of the excitation. Because the excitation of SEHRS occurs by two photons of low energy, and the hyper Raman light is shifted relative to the second harmonic, the energy of both differs significantly. As an example, excitation of HRS with light of a wavelength in the visible results in HRS spectra in the UV (Wen and Hiramatsu, 2020), or with a laser in the near-infrared in the visible wavelength range (Kneipp et al., 1993). As a consequence, an electromagnetic enhancement in SEHRS by plasmonic nanostructures would ideally require the possibility of resonances with localized surface plasmons (Hao and Schatz, 2004) in both wavelength regions. In principle, this can be attained with aggregates of nanoparticles, or also with nanorods or custom made double-resonant antenna structures (Myroshnychenko et al., 2008; Okamoto and Imura, 2013; Butet and Martin, 2015).

Silver nanostructures have been used for SEHRS experiments with great success since its early days (Baranov and Bobovich, 1982; Golab et al., 1988; Kneipp et al., 1993; Itoh et al., 2006; Leng et al., 2006; Itoh et al., 2009). Nevertheless, the high biocompatibility of gold nanostructures has raised our particular interest in exploiting gold nanoparticles (Kneipp et al., 2006a; Živanović et al., 2017), gold containing composite nanostructures (Madzharova et al., 2019a), as well as solid nanosubstrates of gold (Madzharova et al., 2019b) for potential bioanalytical applications of SEHRS. Moreover, SEHRS spectra have been successfully excited in the short-wave infrared (SWIR) range (Turley and Camden, 2014; Simmons et al., 2015), including a recent report on SEHRS excitation off-resonance with molecular electronic transitions in cell cultures samples (Heiner et al., 2021). In a SEHRS experiment in a biological sample, the excitation conditions, as well as the interaction with the plasmonic substrate and its biocompatibility play a major role.

Despite a large number of experiments where the excitation light may be in two-photon electronic resonance in the molecule undergoing the HRS process (Gühlke et al., 2016; Wen and Hiramatsu, 2020), several recent experiments suggest the feasibility of non-resonant SEHRS. This necessitates the discussion of the influence of the excitation wavelength on the electromagnetic field enhancement of SEHRS generated by gold nanostructures of a defined geometry, which is the aim of this paper.

The selection of dimers of gold nanospheres and gold nanorods with a fixed size and inter-particle gap is based on a previous systematic experimental study, where we explored SEHRS enhancement by gold nanostructures as a function of nanoparticle size using an excitation wavelength of 1,064 nm, as well as finite-difference time-domain (FDTD) simulations of local field enhancements for this specific excitation condition (Madzharova et al., 2018). While the distribution of the local fields around typical gold nanostructures based on FDTD data has been studied before, the discussion of two fields occurring at very different wavelengths for such structures has implications for the electromagnetic enhancement that can be obtained in SEHRS. As will be shown by a separate discussion of the contributions from an enhanced excitation intensity and an enhanced HRS intensity, excitation in the SWIR wavelength range is extremely versatile, particularly due to a strong enhancement of the HRS field intensities that contribute linearly to the overall SEHRS enhancement. The results have implications for the design of efficient and biocompatible non-resonant probing by SEHRS.

## Materials and Methods

For the FDTD simulations Lumerical software (FDTD Solutions) was used. The size of the spheres and of the rods was the same in all three-dimensional simulations. The diameter of the gold nanospheres was 44 nm. The thickness of the rods was 16 nm, the length was 80 nm, corresponding to an aspect ratio of 5. The gap between the nanostructures was kept constant at 2 nm. Additional simulations using an excitation wavelength of 1,550 nm were carried out for 70 nm gold nanospheres and single gold nanorod of a length of 80 nm and a diameter of 16 nm. The refractive index of the surrounding medium was 1.33, corresponding to an aqueous medium, as e.g., a solution of biomolecules. Plane wave excitation was used, with the plane wave propagating in opposite z-direction as indicated in the plots. The plane wave was polarized in x-direction, that is, along the long axis of the dimers. Boundary conditions in x- and y-direction were chosen as periodic, and in z-direction as perfectly-matched layer (PML). A mesh size of 0.5 nm was chosen in each simulation and for the whole volume, representing a good balance of the accuracy and the computational time for nanostructures of this geometry (Madzharova et al., 2018). The frequency dependent dielectric constants of gold were used from ref. (Johnson and Christy, 1972). Simulations were carried out for 18 sets of wavelengths, each containing an excitation wavelength of the laser light and a second one corresponding to the Stokes HRS of a 1,586 cm^−1^ ring stretching mode of an organic molecule.

Fields E_ex_ and E_HRS_ were normalized by the incident field E_0_, at the frequency of the excitation field and the hyper Raman Stokes field, respectively. The SEHRS intensity enhancement was calculated as |E_ex_|^4^|E_HRS_|^2^ with E_ex_ being the local field enhancement at the excitation wavelength and E_HRS_ the field enhancement at the HRS wavelength (Kneipp et al., 1993).

Absorbance spectra were simulated in a wavelength range of 400 –2,500 nm, using 250 wavelengths. FDTD extinction was calculated for a nanosphere dimer, a single gold nanorod and a nanorod dimer using the extreme polarization angles parallel and perpendicular to the x-z-plane (defined by the k-vector and the long axis of the dimer).

## Results and Discussion

### Electromagnetic Enhancement of the SEHRS as a Function of Excitation Wavelength

From the enhancement of the excitation field and the enhancement of the hyper Raman scattering (HRS) field, the electromagnetic enhancement factor for the SEHRS process can be calculated as product of the intensity increase in each of the involved fields, that is, |E_ex_|^4^|E_HRS_|^2^ (Kneipp et al., 1999; Kneipp et al., 2006a). We used finite-difference time-domain (FDTD) simulations to study the distribution of the electric field in the proximity of dimers of spherical gold nanoparticles of 44 nm diameter, and of gold nanorods of 80 nm length and 16 nm thickness with a fixed distance between the nanoparticles in all dimers of 2 nm. These parameters were chosen based on previous work, where we discussed the enhancement of SEHRS as a function of nanosphere size using experimental and simulation data (Madzharova et al., 2018). The field enhancement was computed for wavelengths across the range from 540 to 1,800 nm that has been accessed in typical Raman and hyper Raman experiments so far (Atsunari et al., 1979; Kneipp et al., 1993; Turley and Camden, 2014; Simmons et al., 2015; Madzharova et al., 2018; Heiner et al., 2021). As HRS wavelength for each excitation wavelength, we used the wavelength of the hyper Raman scattered light corresponding to the frequency of a typical phenyl ring stretching vibration, that could occur in various molecules. Note that contributions by chemical enhancement to the SEHRS, which can be significant (Golab et al., 1988; Valley et al., 2010), as well as potential two-photon electronic resonance in a molecule (Gühlke et al., 2016; Madzharova et al., 2018), are completely neglected here.

**FIGURE 1:**
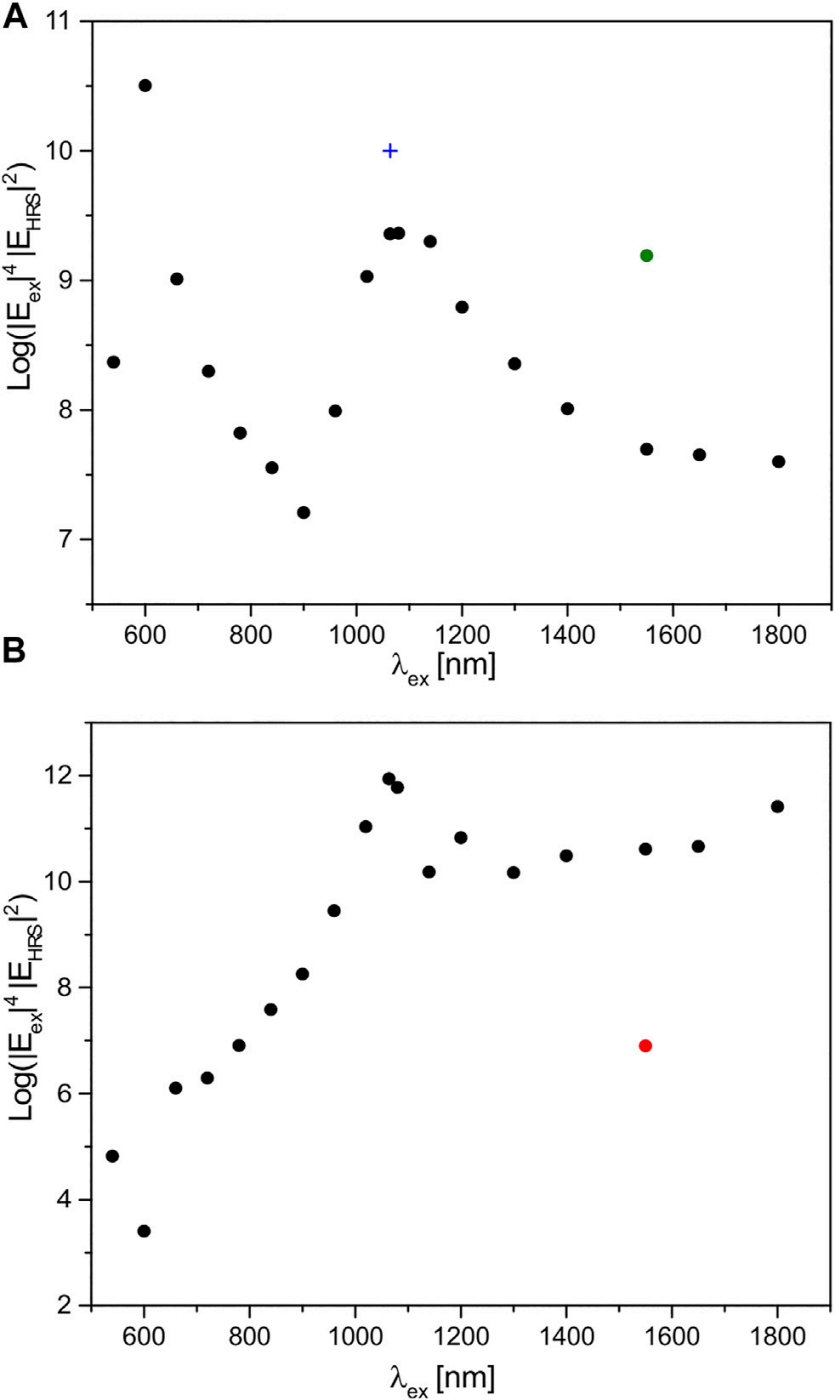
Maximum SEHRS enhancement, as a function of excitation wavelength, as a result of 3D FDTD simulations in an aqueous environment of a dimer **(A)** of gold nanospheres of a diameter of 44 nm and **(B)** of gold nanorods with a length of 80 nm and a thickness of 16 nm. The maximum enhancement was determined in a 2D map of the SEHRS intensity in the equatorial plane of the 3D structure. The gap between the particles is 2 nm. The propagation of the incident plane wave is in z direction, and it is polarized along the long axis of the dimers (cf. Figure 3 or Figure 4). The green dot in **(A)** indicates the SEHRS enhancement of a dimer of gold nanospheres with a diameter of 70 nm, corresponding to the geometry of nanospheres in the experiments reported in ref. (Heiner et al., 2021). The blue cross in **(A)** marks the SEHRS enhancement of a dimer of gold nanospheres with a diameter of 60 nm diameter reported by ref. (Madzharova et al., 2018). The red dot in **(B)** indicates the maximum SEHRS enhancement obtained in the simulation using a single gold nanorod of the same dimensions instead of the nanorod dimer. The SEHRS enhancement is the product of the normalized electric field intensity at the excitation wavelength and the respective Stokes HRS wavelengths chosen here to correspond to a molecular vibration with a wavenumber of 1,586 cm^−1^.

In Figure 1, the maximum enhancement is shown as a function of excitation wavelength for the dimers of nanospheres (Figure 1A) and nanorods (Figure 1B). The maximum SEHRS enhancement obtained with the dimer of gold nanospheres varies by three to four orders of magnitude depending on the wavelength that is chosen for the experiments (Figure 1A). Two peaks are observed in the plot, one in the visible wavelength region around 600 nm and another one at the excitation wavelengths of 1,064 and 1,080 nm. The strong enhancement by the gold nanosphere dimer at an excitation of 1,064 nm is in agreement with successful experiments using this wavelength with gold nanoparticles in previous work (Madzharova et al., 2018). In earlier simulations, we found a strong dependence of SEHRS enhancement on nanoparticle size, enabling its further increase with increased diameter of the gold nanospheres at equal interparticle spacing (Madzharova et al., 2018). As an example, the SEHRS enhancement was higher by almost one order of magnitude in nanodimers of 60 nm spheres (Madzharova et al., 2018) (Figure 1A, blue cross), compared to the 44 nm particles studied here.

Figure 1B shows the maximum SEHRS enhancement provided by the dimer of gold nanorods. It varies by nine orders of magnitude across the applied range of excitation wavelengths, much more than the enhancement observed by the gold nanospheres (cf. Figure 1A). At an excitation wavelength of 1,064 nm, the SEHRS enhancement of the rods reaches a value of 10^11^, as reported already previously (Madzharova et al., 2018). Figure 1B here shows that this is the maximum enhancement that can be obtained with a gold nanorod dimer of this geometry across the applied wavelength range, and that a laser operating at 1,064 nm is an ideal excitation source for experiments with such gold nanostructures.

### Contributions by Excitation and HRS Fields

**FIGURE 2:**
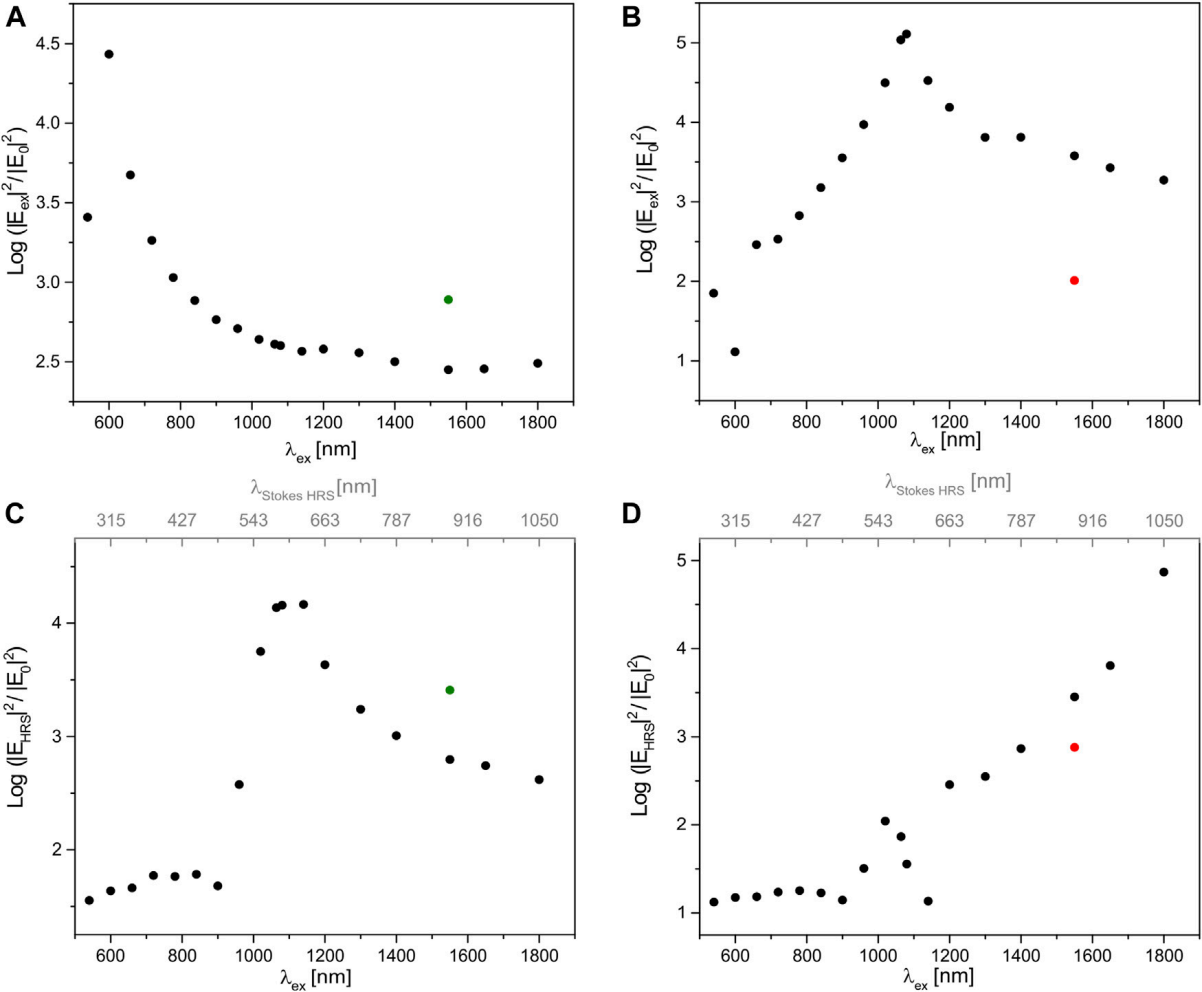
Maximum intensity enhancement of **(A,B)** the normalized excitation field and **(C,D)** the normalized field of the hyper Raman scattering as a function of excitation wavelength provided by a dimer **(A,C)** of gold nanospheres of a diameter of 44 nm and **(B,D)** of gold nanorods with a length of 80 nm and a thickness of 16 nm as a result of 3D FDTD simulations. Values on the top axes in **(C)** and **(D)** represents the Stokes HRS wavelengths corresponding to a molecular vibration with a wavenumber of 1,586 cm^−1^ for the excitation wavelength indicated on the; bottom axis. The gap between the particles is 2 nm. The propagation of the incident plane wave is in z-direction, and it is polarized along the long axis of the dimers (cf. Figure 3 or Figure 4). The green dots in **(A)** and **(C)** indicate the intensity enhancements of a dimer of gold nanospheres with a diameter of 70 nm, corresponding to the geometry of nanospheres in the experiments reported in ref. (Heiner et al., 2021). The red dots in **(B)** and **(D)** indicate the maximum intensity enhancements attained by of a single gold nanorod of the same dimensions.

As the enhancement of SEHRS intensity is the result of an intensity enhancement of the excitation field and of the hyper Raman Stokes field, which each correspond to wavelengths in a completely different spectral range, both must be discussed separately in order to connect SEHRS enhancement to the optical properties of the gold nanodimers. Figure 2 shows the maximum field intensity enhancements of the excitation field |E_ex_/E_0_|^2^ (Figures 2A,B, respectively) and of the Stokes HRS field |E_HRS_/E_0_|^2^ (Figures 2C,D, respectively) for the dimers of nanospheres and nanorods. In Figure 2 we can compare which field dominates the overall enhancement at which excitation wavelength. Due to the quadratic contribution by the excitation intensity, that is |E_ex_|^4^, to the SEHRS intensity (Kneipp et al., 1993), it seems obvious that the excitation field may simply outweigh the contribution of the enhancement of the HRS field intensity |E_HRS_|^2^. In agreement with this, the SEHRS intensity enhancement provided by the nanosphere dimer follows the course of the enhancement of the excitation field shown in Figure 2A in the range between 540 and 900 nm (compare Figures 1A, 2A). Similarly, in the dimer of gold nanorods, the increasing SEHRS enhancement (Figure 1B) follows the growth of the intensity of the excitation field up to an excitation wavelength of ∼1,100 nm (Figure 2B).

As visible in the dependency of SEHRS intensity enhancement (Figure 1) and the intensity of the HRS field alone (Figures 2C,D) in the short-wave infrared range, the HRS intensity enhancement can become very high as well and compensate the weaker enhancement of the excitation intensity in that range (Figures 2A,B). In the gold nanosphere dimers, this is observed for an excitation wavelength around 1,000 nm and higher, including the high SEHRS enhancement observed with 1,064 nm excitation discussed above (compare Figure 2C with Figure 1A). Clearly, the strong enhancement of the HRS intensity at HRS wavelengths around and above 540 nm (cf. top scale of Figure 2C) is responsible here (compare Figure 2C with Figure 1A). In the nanorod dimers, the high SEHRS enhancement with excitation ∼1,800 nm (Figure 1B) is caused by high intensities of the HRS field for HRS wavelengths approaching ∼1,000 nm (Figure 2D, read top scale). The increase in HRS field intensity across four orders of magnitude (Figure 2D) compensates well the decrease of the excitation intensity by two orders of magnitude in the range from 1,080 to 1,800 nm (Figure 2B), as the latter contributes with second power to SEHRS intensity enhancement (Figure 1B), which therefore remains more or less at the same level of ∼10^10^ between 1,100 and 1,800 nm (Figure 1B). Similarly, the very low HRS enhancement for an excitation wavelength of 1,140 nm, corresponding to an HRS wavelength of ∼600 nm (Figure 2D), influences the high enhancement of the excitation field (Figure 2B), causing a “dip” in the SEHRS enhancement at 1,140 nm (Figure 1B). The importance of an enhanced HRS field to the SEHRS intensity enhancement has been discussed previously for an aluminum dipolar antenna working for wavelengths around 400 nm (Butet and Martin, 2015).

It should be pointed out that the electromagnetic enhancement of the (one-photon) process of SERS, approximated by |E_ex_|^4^ due to the similarity of |E_ex_|^2^ and the Raman Stokes intensity |E_RS_|^2^, directly follows the dependency of the excitation field enhancement (Supplementary Figure S1). Thereby, it differs from the SEHRS enhancement, especially for excitation wavelengths above 900 nm in the dimer of gold nanospheres (compare Figure 1A with Supplementary Figure S1A) and above ∼1,100 nm in the gold nanorods (compare Figure 1B with Supplementary Figure S1B). The maximum enhancement yielded with a dimer of spheres shows a prominent enhancement maximum at 600 nm (Supplementary Figure S1A), with a dimer of nanorods around 1,100 nm (Supplementary Figure S1B).

In summary, the decrease of the enhancement of the SEHRS intensity in the gold nanosphere dimer repeats twice with increasing excitation wavelength: for excitation wavelengths from ∼540 to 900 nm (Figure 2A), and again for the HRS wavelengths from ∼540 to ∼1,000 nm (Figure 2C, cf. top scale), with a maximum around 600 nm, and an overlap of the profiles of the two fields. The maximum in enhancement for both fields is in agreement with the extinction spectra of gold nanospheres, which show an absorbance at 540 nm in experiments (Madzharova et al., 2018) (cf. Supplementary Figure S2A for simulated spectra).

In the nanorod dimer, the field intensity enhancement is clearly determined by the absorbance of the longitudinal plasmon mode (Link and El-Sayed, 1999), located around 900 nm for nanorods of this aspect ratio (Myroshnychenko et al., 2008; Madzharova et al., 2018) (see also Supplementary Figure S2B for simulated spectra). Also following the steep decrease of the real part of the gold dielectric function (Johnson and Christy, 1972), the increase of the intensities of the excitation field (Figure 2B) and the HRS field (Figure 2D, cf. top scale) from ∼700 nm toward ∼1,000 nm is seen. For wavelengths above 1,100 nm, the excitation intensity enhancement decreases by one or so order of magnitude (Figure 2B), in agreement with damping setting in (Johnson and Christy, 1972), albeit not as much as observed in the nanosphere dimer (Figure 2A), due to different geometries and contributions by different damping mechanisms (Sönnichsen et al., 2002; Myroshnychenko et al., 2008; Derkachova et al., 2016).

The range of shorter wavelengths in the visible has not been emphasized here with respect to excitation, due to lower biocompatibility (photo damage) and the fact that the corresponding HRS wavelengths lie in the UV spectral region. Nevertheless, the intensity enhancements of excitation and HRS light below and around 600 nm must be discussed, as they are clearly related to the transversal plasmon mode in the nanorods (Link and El-Sayed, 1999) here that was found to display a maximum around 515 nm in experiments (Madzharova et al., 2018) and that is also observed in this range in a simulation (cf. Supplementary Figure S2B). In this context, the small maximum excitation field enhancement at a wavelength of 600 nm (Figure 2B) is not an “outlier” in a row of ascending values, but it marks the transition between an enhancement obtained due to resonance with the transversal mode (Supplementary Figure S3), also indicated in the profile of the hyper Raman field (cf. top axis of Figure 2D), and the increasing contribution by the longitudinal resonance above 600 nm. This is the same for the HRS field enhancement (Figure 2D, read top scale), where the field intensity of the HRS scattering raises for an excitation wavelength of 960 nm and becomes a local maximum at 1,020 nm that corresponds to an HRS wavelength of ∼540 nm, in resonance with the transversal mode. For HRS wavelengths above 600 nm, the resonance with the longitudinal mode takes over.

### Distribution of the SEHRS Intensity Enhancement and of the Enhancement of the Separate Field Intensities

**FIGURE 3:**
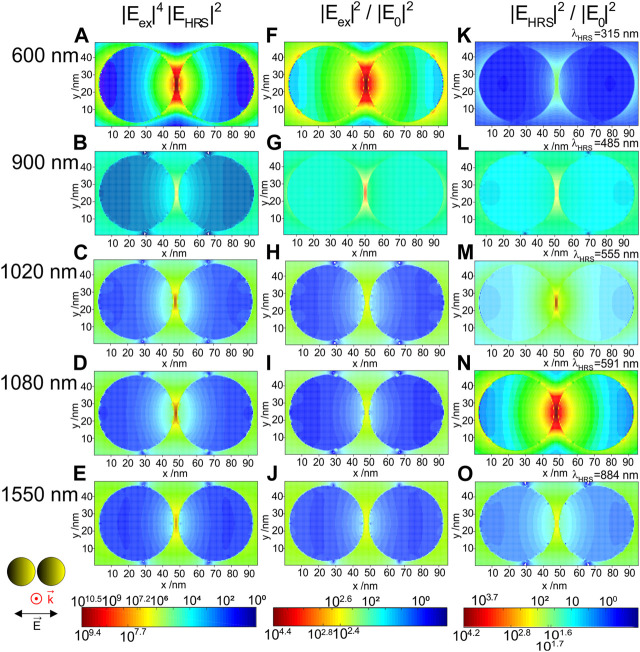
**(A–E)** SEHRS enhancement of a gold nanosphere dimer and corresponding normalized field intensities at **(F–J)** excitation wavelengths, and at **(K–O)** Stokes HRS wavelengths in the x,y-plane as result of 3D FDTD simulations. The diameter of the gold nanospheres is 44 nm. The gap between the particles is 2 nm. The propagation and polarization of the incident plane wave are indicated. The monitor is placed in the equatorial plane. The wavelength of the HRS for each of the excitation wavelengths corresponds to a molecular vibration with a wavenumber of 1,586 cm^−1^ and is indicated in each of the panels **(K)** to **(O)**. The maximum SEHRS enhancement of 10^10.5^ is found for an excitation wavelength of 600 nm **(A)** The highest excitation intensity enhancement, also observed at 600 nm has a value of 10^4.4^ **(F)**. The maximum HRS intensity enhancement of 10^4.2^ is obtained for a Stokes HRS wavelength of 591 nm, upon excitation at 1,080 nm **(N)**.

The 2D intensity distribution maps reflect well the contributions by the enhancement of the excitation field and the HRS field. Figure 3 displays the spatial distribution of the field around the dimer of gold nanospheres for selected excitation wavelengths at which high enhancements were found for the SEHRS intensity and/or for excitation or HRS intensities. The maximum SEHRS enhancement of 10^10.5^ that is found at an excitation wavelength of 600 nm (Figure 2A) occurs in the hotspot that forms in the inter-particle gap, in full agreement with all previous discussions (Blatchford et al., 1982; Le et al., 2008; McMahon et al., 2011; Stockman, 2011; Kadkhodazadeh et al., 2013; Kneipp et al., 2015). It decreases drastically for an excitation at 900 nm (Figure 3B), because of the very low contribution by the HRS field at the corresponding HRS wavelength (Figure 3L). In the dimer geometry shown here, the SEHRS intensity enhancement has a second maximum (Figure 1A), about one order of magnitude lower for excitation wavelengths around 1,080 nm (Figures 3C,D), before it decreases to ∼10^8^ (Figure 3E, see also Figure 1A). As discussed above (cf. Figure 2C), the high SEHRS enhancement observed for excitation around 1,020 and 1,080 nm (Figures 3C,D) is caused by high HRS field intensities (Figures 3M,N, respectively). When investigated for spheres of different diameter, the strong enhancement of the hyper Raman field for an excitation at 1,064 nm does not vary much with the size of the nanospheres in the dimer (Madzharova et al., 2018), different from high sensitivity of field enhancement with respect to interparticle spacing (Nordlander et al., 2004; Dhawan et al., 2009; McMahon et al., 2011; Kadkhodazadeh et al., 2013).

**FIGURE 4:**
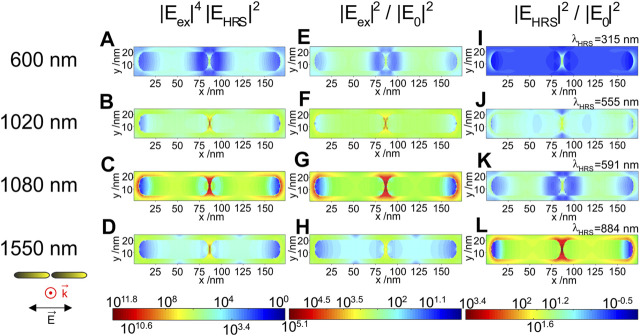
**(A–D)** SEHRS enhancement of a gold nanorod dimer and corresponding normalized field intensities at **(E,F)** excitation wavelengths, and at **(I–L)** Stokes HRS wavelengths in the x,y-plane as result of 3D FDTD simulations. The length of the rods is 80 nm and their thickness is 16 nm. The gap between the particles is 2 nm. The propagation and polarization of the incident plane wave are indicated. The monitor is placed in the equatorial plane. The wavelength of the HRS for each of the excitation wavelengths corresponds to a molecular vibration with a wavenumber of 1,586 cm^−1^ and is indicated in each of the panels **(I)** to **(L)**. The maximum SEHRS enhancement of 10^11.8^ is found for an excitation wavelength of 1,080 nm **(C)**. The highest excitation intensity enhancement, also observed at 1,080 nm has a value of 10^5.1^ **(G)**. The maximum HRS intensity enhancement of 10^3.4^ is obtained for a Stokes HRS wavelength of 884 nm, upon excitation at 1,550 nm **(L)**.

The spatial distribution of the SEHRS enhancement provided by the dimer of the gold nanorods (Figures 4A–D) also shows the formation of a hot spot in the inter-particle gap. As discussed above (Figure 1B), the maximum SEHRS enhancement of the gold nanorods of ∼10^11^ is found for excitation with a wavelength of 1,080 nm, and in good agreement with previous results from experiments and simulations (Madzharova et al., 2018). The high enhancement of the excitation field intensity is responsible here (Figures 2B, 4G), while the enhancement of the hyper Raman light is not supported by the longitudinal plasmon mode. HRS intensity relies on the transversal mode that cannot be efficiently excited with the polarization of the electric field along the long axis of the nanorod dimer, contributing a very weak enhancement by a factor of only ∼10 (Figure 4K). The responsibility of the transversal mode to generate the field enhancement for the HRS here is also illustrated quite well in the HRS intensity distribution obtained with excitation at 1,020 nm (Figure 4J), causing a resonance of the HRS frequency with the mode around 540 nm. There, regions of higher intensity are observed along the long sides of the two rods, very different from the field concentration found at the tips of the nanorods and in the gap when excitation or HRS wavelength are in resonance with the longitudinal mode (compare Figures 4J,K with Figures 4F,G,L).

The high intensities in the gap between the nanorods indicate efficient coupling of the longitudinal plasmon modes of both nanorods in the studied geometry and excitation condition (Funston et al., 2009), as is the case in resonance with the excitation at 1,080 nm (Figure 4G) or with the HRS wavelength of 884 nm, upon excitation at 1,550 nm (Figure 4L). Moreover, comparison of the maximum SEHRS enhancement that can be obtained with an individual nanorod for an excitation wavelength of 1,550 nm and an HRS wavelength of 884 nm (Supplementary Figure S4) shows that the coupling becomes even more important for the longer excitation wavelength rather than the HRS wavelength (see also Figures 2B,D, red marking). The enhancement of the HRS intensity at 884 nm is only weaker by less than a factor of three in the single rod (compare Supplementary Figure S4C with Figures 2D, 4L, red marking). In contrast, the enhancement of the excitation field (Figure 2C, red marking, Supplementary Figure S4B) is lower by one to two orders of magnitude in the single nanorod, indicating that in the axial nanorod dimers plasmon coupling must lead to resonances with the long excitation wavelengths (Aizpurua et al., 2005; Willingham et al., 2008; Funston et al., 2009). Furthermore, though not accounted for in the discussion of the plasmon resonances here, the lightning rod effect can become efficient, especially for the long excitation wavelengths (Liao and Wokaun, 1982; Aizpurua et al., 2005; Le et al., 2008).

### Implications for SEHRS in Biocompatible Settings

In agreement with the dependence of the decaying field on a distance *r* with *1/r* ^*3*^ and hence the intensity for each of the single fields with *1/r* ^*6*^, the SEHRS intensity enhancement (Figures 3A–D, 4A–D) as a product of the non-linear excitation and the hyper Raman field intensities yields an extreme confinement of the regions of high SEHRS enhancement in the hot spots. Although the product of all fields must be considered, the contributions by the excitation field and the hyper Raman field can differ greatly both in magnitude as well as in distribution (compare middle and right columns in Figures 3, 4). This also has consequences for the extension of the regions of very high local fields, located here in the gap between the particles in both kinds of dimers. Supplementary Figure S5 shows the derivative of the intensity along the short axis of the dimer in the center of the hot spot, providing an idea of both the extension and the decay of the field intensity in that particular direction. As expected, the decrease of the SEHRS intensity enhancement is very steep (Supplementary Figures S4D, S5A), with much stronger confinement than for the individual fields, compare for example the change in the SEHRS enhancement at 600 nm in the nanospheres (Supplementary Figure S5A, black curve and Figure 3A) with that in the corresponding excitation intensity (Supplementary Figure S5B, black curve and Figure 3E). As another example, the field of the HRS light excited in the SWIR in the nanorod dimer, with 1,550 nm (Supplementary Figure S5F, magenta curve), extends very far, with a very steep decay around 2.5 nm from the hot spot. It must be noted here that resolution in the maps is defined by the mesh size of 0.5 nm, and that the model used here does not consider several specifics of the hot spot that have been discussed in more elaborate theoretical works based on other approaches (McMahon et al., 2011; Esteban et al., 2012), including the observation of cascade effects at atomistic length scales (Barbry et al., 2015), that can be come very important.

The extension of the hot spots has consequences for real SEHRS experiments. Especially for experiments with “large” macromolecules, there are implications, due to their size, preventing them to fit in a gap of 2 nm (Erickson, 2009), but moreover for the very local probing of those parts of the molecule that are in immediate proximity to the gold nanostructure due to decay of the enhancement. In the biomolecular context, such spatial selectivity, related to confinement of the highest enhancements to the hottest hot spots was also reported for the highly selective probing by SERS, observed e.g., in the SERS spectra of proteins (Xu et al., 1999), where it enables selective probing of interaction sites (Szekeres and Kneipp, 2018). For large-molecule characterization by SEHRS it would be beneficial to exploit a large area of high enhancement distribution as an extension of the hotspot. In this regard, utilization of the tips of nanorods appears more useful than the interparticle gap. To assess the properties of the “hot spots,” local probing at nanoscopic resolution by electron energy loss spectroscopy can provide a better idea about the actual situation in an experiment with nanoparticles, and can be combined with an estimate of the enhancement from a combined SEHRS and SERS experiment, as has been demonstrated for silver dimers previously (Kadkhodazadeh et al., 2013). In these (Kadkhodazadeh et al., 2013), and other experiments (Kumari et al., 2015), strong influence by the gap size, observed in simulations (Nordlander et al., 2004; Dhawan et al., 2009; McMahon et al., 2011) was confirmed as well.

In SEHRS or SERS experiments with nanoparticles and biomolecules, the analyte molecules themselves often can function as spacers within the nanoaggregates, determining their plasmonic properties (Funston et al., 2009; Madzharova et al., 2018). This renders particular enhancement quite vulnerable to the specific experimental situation. The enhancements discussed for the nanorods here for excitation in the SWIR that is necessary in the biological context due to lower photo damage, appear to be very robust with respect to excitation wavelength (Figure 1B). As mentioned, this wavelength range is also particularly attractive due to the possibility that other contributions such as the lightning rod effect can support the enhancement.

Although it is not discussed here in detail for this frequency range, apart from having the real environment influencing the enhancement, decisive tuning of the size of spherical nanoparticles can improve the enhancement of the nanosphere dimers to the level of that of the nanorod dimers. As an example simulated here, Figure 1A (green dot) indicates the SEHRS enhancement that is obtained with a dimer of larger spheres of a diameter of 70 nm with excitation at 1,550 nm, also with a significant contribution by resonance with the HRS wavelength (Figure 2C, green dot). Applicability in bioprobing was confirmed by successful experiments with this type of nanostructure in live cells recently (Heiner et al., 2021).

## Conclusion

In summary, probing the electromagnetic field enhancement in SEHRS across a broad range of excitation wavelengths confirms that the enhancement of the intensity of the laser field used for excitation is very important due to its non-linear contribution. Nevertheless, as shown by the separate analysis of the intensity enhancement of both fields, the excitation and the HRS radiation, the hyper Raman Stokes field enhancement can take over a relatively stable control of the SEHRS enhancement for excitation wavelengths longer than 1,000 nm. For the gold nanostructures that were studied here, the matching of LSPR with typical HRS wavelengths is available across the whole SWIR wavelength range, with less variation/decrease towards long wavelengths for the nanorods. As was discussed, this can be related to different coupling behavior and decay of the LSPR in the different nanostructures. The possibility to control both, excitation wavelength and nanoparticle size, will enable robust SEHRS bioprobing using excitation in the SWIR wavelength range even in highly variable biomolecular environments that exert an influence on nanostructure geometry and interparticle spacing.

## Data Availability

The raw data can be made available upon reasonable request.
