# Supplementary material for: Excitation Conditions for Surface-Enhanced Hyper Raman Scattering With Biocompatible Gold Nanosubstrates
Source: Front Chem. 2021 May 17;9:680905. doi: 10.3389/fchem.2021.680905 (PMC8165379; doi:10.3389/fchem.2021.680905)
Supplement: Supplementary file 1 [file DataSheet1.PDF]

Supplementary Material

Excitation conditions for surface-enhanced hyper Raman scattering with biocompatible gold nanosubstrates

Arpad Dusa, Fani Madzharova, Janina Kneipp*

Humboldt-Universität zu Berlin, Department of Chemistry, Brook-Taylor-Str. 2, 12489 Berlin, Germany

*corresponding author: janina.kneipp@chemie.hu-berlin.de

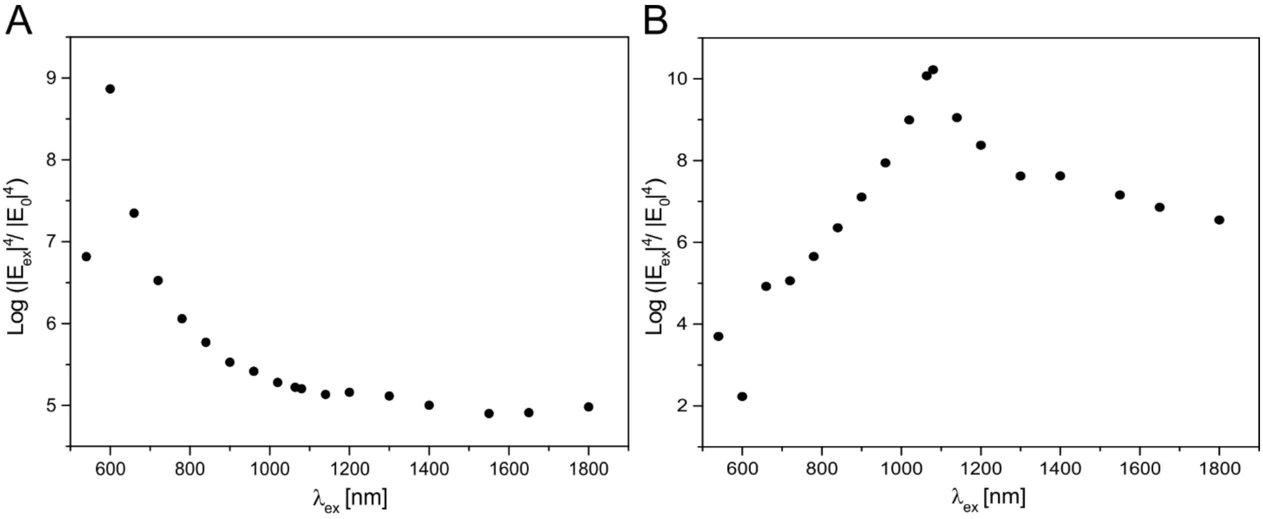

Figure S1. Maximum SERS enhancement as a function of excitation wavelength, as a result of 3D FDTD simulations in an aqueous environment of a dimer **(A)** of gold nanospheres of a diameter of 44 nm and **(B)** of gold nanorods with a length of 80 nm and a thickness of 16 nm. The maximum enhancement was determined in a 2D map of the SERS intensity in the equatorial plane of the 3D structure. The gap between the particles is 2 nm. The propagation of the incident plane wave is in z direction, and it is polarized along the long axis of the dimers (cf. Figure 3 or Figure 4). The SERS enhancement is the product of the normalized electric field intensity at the excitation wavelength and the respective Stokes wavelength, approximated here by the square of the excitation field.

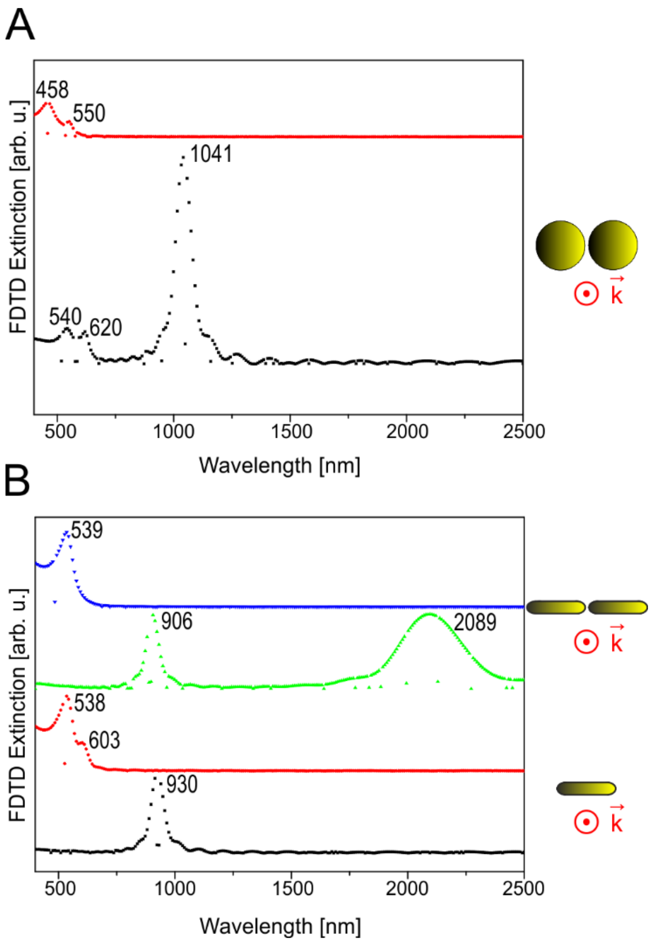

Figure S2. FDTD simulation of extinction spectra in the wavelength range of 400 nm to 2500 nm for **(A)** a dimer of gold nanospheres and **(B)** gold nanorods. The schematics on the right hand side illustrate the alignment of the nanostructure and the propagation direction of the excitation plane wave. The diameter of the gold nanospheres is 44 nm, the length of the rods 80 nm and the thickness 16 nm. In each case, the gap between the particles is 2 nm. In order to The two respective spectra show the results for the extreme polarization angles parallel and perpendicular to the x-z-plane (defined by the k-vector and the long axis of the dimer).

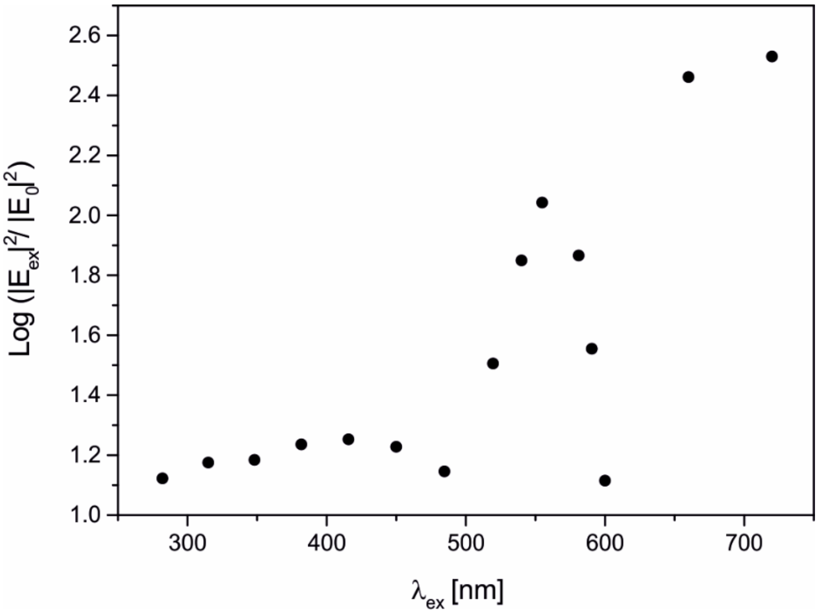

Figure S3. Maximum intensity enhancement of the normalized excitation field as a function of excitation wavelength from UV range to 720 nm, provided by dimer of gold nanorods with a length of 80 nm and a thickness of 16 nm as a result of 3D FDTD simulations.

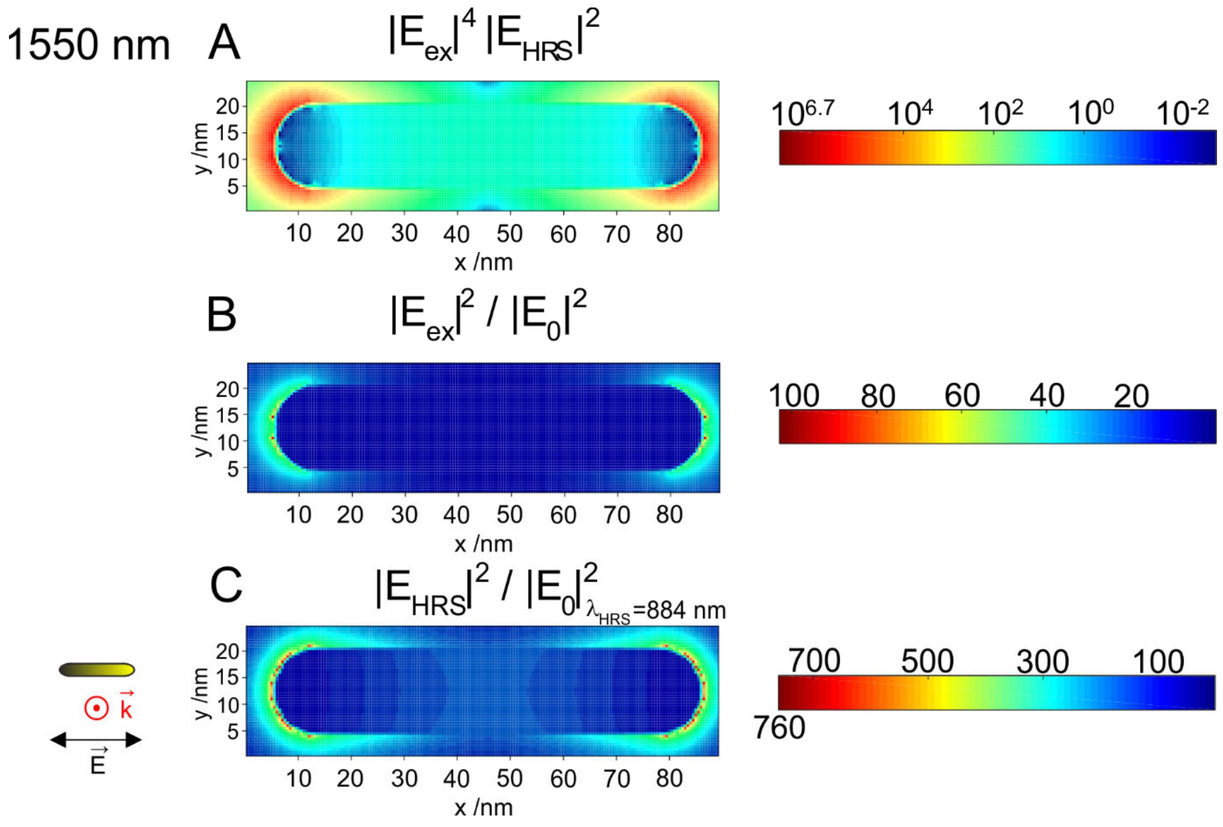

Figure S4. (A) SEHRS enhancement calculated from the normalized field intensities **(B)** for an excitation wavelength of 1550 nm, and a **(C)** Stokes HRS wavelength of 884 nm in the x,y-plane as result of a 3D FDTD simulations. The length of the gold nanorod is 80 nm, its thickness is 16 nm. The schematic at the bottom left corner indicates the alignment of the single nanorod, the propagation of the incident plane wave, and the polarization.

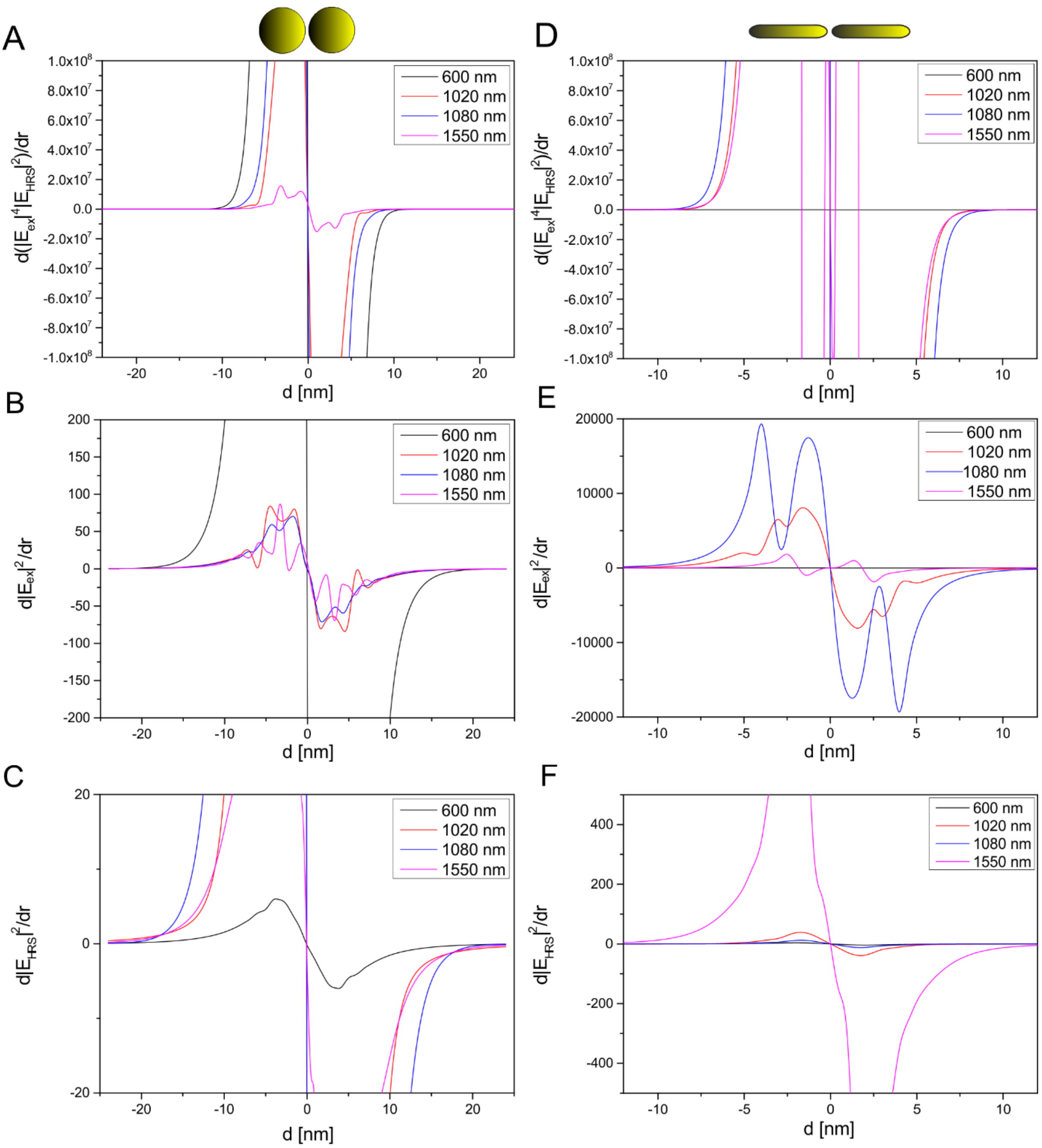

Figure S5. First derivative of the enhancement profile along the y-direction in the center of the inter-particle gap for the gold nanosphere dimer of **(A)** the SEHRS enhancement, of **(B)** the intensity enhancement of the excitation field, of **(C)** the intensity enhancement of the HRS field for the four indicated wavelengths. The according 2D maps are shown in Figure 3 of the manuscript. First derivative of the enhancement profile along the y-direction in the center of the inter-particle gap for the gold nanorod dimer of **(D)** the SEHRS enhancement, of **(E)** the intensity enhancement of the excitation field, of **(F)** the intensity enhancement of the HRS field for the four indicated wavelengths. The according 2D maps are shown in Figure 4 of the manuscript. The diameter of the gold nanospheres was 44 nm, the length of the gold nanorods 80 nm and their thickness 16 nm. The gap between the particles was 2 nm.
